# Nuclear and Mitochondrial Genome Assemblies for the Endangered Wood-Decaying Fungus *Somion occarium*

**DOI:** 10.1093/gbe/evaf003

**Published:** 2025-01-10

**Authors:** Rowena Hill, Jamie McGowan, Vendula Brabcová, Seanna McTaggart, Naomi Irish, Tom Barker, Vanda Knitlhoffer, Sacha Lucchini, Kendall Baker, Leah Catchpole, Chris Watkins, Karim Gharbi, Gemy Kaithakottil, Alan Tracey, Jonathan M D Wood, Michal Tomšovský, Petr Baldrian, David Swarbreck, Neil Hall

**Affiliations:** Earlham Institute, Norwich, UK; Earlham Institute, Norwich, UK; Laboratory of Environmental Microbiology, Institute of Microbiology of the Czech Academy of Sciences, Prague, Czech Republic; Earlham Institute, Norwich, UK; Earlham Institute, Norwich, UK; Earlham Institute, Norwich, UK; Earlham Institute, Norwich, UK; Earlham Institute, Norwich, UK; Earlham Institute, Norwich, UK; Earlham Institute, Norwich, UK; Earlham Institute, Norwich, UK; Earlham Institute, Norwich, UK; Earlham Institute, Norwich, UK; Tree of Life, Wellcome Sanger Institute, Hinxton, UK; Tree of Life, Wellcome Sanger Institute, Hinxton, UK; Department of Forest Protection and Wildlife Management, Faculty of Forestry and Wood Technology, Mendel University in Brno, Brno, Czech Republic; Laboratory of Environmental Microbiology, Institute of Microbiology of the Czech Academy of Sciences, Prague, Czech Republic; Earlham Institute, Norwich, UK; Earlham Institute, Norwich, UK; School of Biological Sciences, University of East Anglia, Norwich, UK

**Keywords:** saprotroph, white-rot, *cerrenaceae*, *polyporales*, fungal conservation

## Abstract

*Somion occarium* is a wood-decaying bracket fungus belonging to an order known to be rich in useful chemical compounds. Despite its widespread distribution, *S. occarium* has been assessed as endangered on at least 1 national Red List, presumably due to loss of old-growth forest habitat. Here, we present a near-complete, annotated nuclear genome assembly for *S. occarium* consisting of 31 Mbp arranged in 11 pseudochromosomes—9 of which are telomere-to-telomere—as well as a complete mitochondrial genome assembly of 112.9 Kbp. We additionally performed phylogenomic analysis and annotated carbohydrate-active enzymes (CAZymes) to compare gene and CAZyme content across closely related species. This genome was sequenced as the representative for Kingdom *Fungi* in the European Reference Genome Atlas Pilot Project.

**Significance**

Wood-decaying fungi are not only the foundation of nutrient cycling in our forests, but also known to produce many medically relevant chemical compounds. As *Somion occarium* is also endangered in at least 1 country, it is doubly important to produce high-quality genomic resources to facilitate study of this species. The sole fungal representative sequenced as part of the European Reference Genome Atlas (ERGA) Pilot Project, this genome assembly leads the way for future sequencing efforts of fungi within ERGA.

## Introduction

**Fig. 1.**
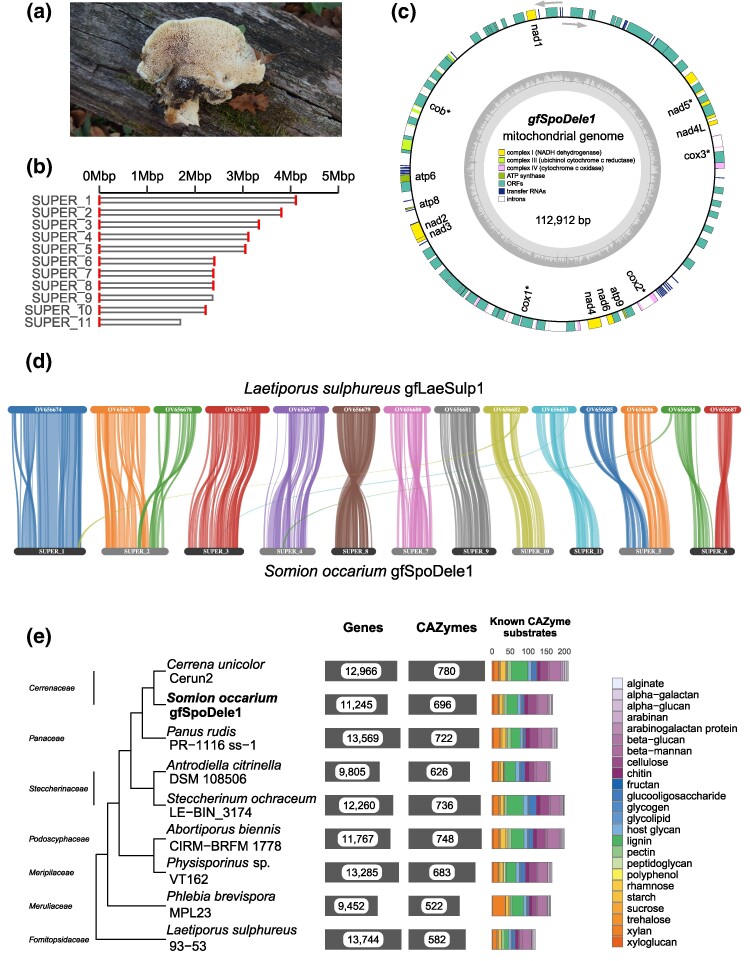
a) Photograph of a *S. occarium* specimen from the same locality as the strain sequenced in this study, taken in 2018. b) A representation of the assembled pseudo-chromosomal contigs, with red bars at each end indicating predicted telomeres. c) The assembled and annotated mitogenome. d) Synteny between *S. occarium* gfSpoDele1 and *L. sulphureus* gfLaeSulp1. e) Comparison of gene content between *S. occarium* and closely related *Polyporales* species, with an emphasis on CAZymes. Known substrates are only shown for CAZyme genes with no contradictory substrate predictions by run_dbcan.

The species *Somion occarium* (*Cerrenaceae*, *Polyporales*, *Basidiomycota*) is a bracket fungus and polypore (Fig. 1a), both of which are polyphyletic groupings based on growth form rather than ancestry. Usually found on dead wood of hardwood trees—including oaks (*Quercus* spp.) and beech (*Fagus* spp.) (GBIF 2024)—*S. occarium* is predominantly a white-rot decayer, or saprotroph. Its occasional presence on living trees suggests it is also capable of facultative parasitic growth, similar to other species in the *Cerrenaceae* (Hallenberg et al. 2008; Justo et al. 2017). In addition to wood, the fungus has also occasionally been recorded from soil DNA metabarcoding (Větrovský et al. 2020). Such wood-decaying fungi are essential for carbon and nutrient cycling and the formation and stabilization of soil aggregates in forest ecosystems (Miller and Lodge 2007).

*Somion occarium* belongs to the order *Polyporales*, which contains many, but not all, of the polypore species. Polypores are iconic in forest ecosystems, and have long been culturally valued, including a rich history of their use in folk medicine (Grienke et al. 2014). Fittingly, the *Polyporales* are some of the highest known producers of bioactive compounds within fungi, including antibacterials, antifungals, and drugs or drug leads (Prescott et al. 2023). Generating genomic data for species in the order provides the groundwork to support further discovery of potentially useful compounds.

The Earth BioGenome Project (EBP) is an ambitious initiative to sequence reference genomes for all eukaryotic species (Lewin et al. 2018). The European Reference Genome Atlas (ERGA) is the European node of EBP, and has coordinated a pilot project to establish and test a decentralized infrastructure to deliver the EBP’s aspirations (Mc Cartney et al. 2024), for which *S. occarium* was selected as a representative for fungi. *Somion occarium* was previously known as *Spongipellis delectans* (or the synonym *Hydnum occarium*), but recent division of *Spongipellis sensu lato* into several genera following DNA-based and morphological revision saw the restoration of the suppressed older genus name *Somion* for the *Sp. delectans* complex (Miettinen et al. 2023). North American *Sp. delectans* is now *Somion delectans*, while European *Sp. delectans* is *S. occarium*. There is 1 existing assembly for the *Cerrenaceae* family, which *S. occarium* belongs to, for the species *Cerrena unicolor* (https://mycocosm.jgi.doe.gov/Cerun2/Cerun2.home.html), although the *S. occarium* ERGA assembly reported here represents the first EBP-level reference genome for the family.

While *S. occarium* is found across the northern temperate zone in Europe and northernmost Africa (Miettinen et al. 2023), the species is classed as endangered on the national Red List for the Czech Republic (Zíbarová et al. 2024), the locality from which the strain sequenced here was collected. Wood-decaying fungi are predominantly dependent on deadwood, which is more available in old-growth forests compared with younger or managed forests/plantations, and so the ongoing loss of old-growth forest habitat means that wood-decaying fungi are increasingly threatened by niche reduction (Jönsson et al. 2008; Lonsdale et al. 2008). Incidentally, this also means that certain fungal species can be used as indicator species for old-growth forest (Halme et al. 2009). Recognition of fungi in conservation discourse is relatively new (May et al. 2018), but progress is being made thanks to the enduring efforts of mycologists to raise their profile in Red Lists (Dahlberg and Mueller 2011; Mueller et al. 2014), as well as growing public and political awareness of the importance of fungi to ecosystems (IUCN 2021). Producing high-quality genomic resources for endangered species such as *S. occarium* is foundational to understanding and protecting them.

## Results and Discussion

### Near-complete Genome Assembly for *S. occarium*

We assembled the *S. occarium* genome using hifiasm (Cheng et al. 2021) from ∼100-fold coverage of PacBio HiFi reads, with a mean read length of 18.6 Kbp. The final, manually curated assembly (Table 1) exceeded the minimum reference standard defined by EBP assembly quality metrics (EBP 2023). Note that this final version of the assembly is a primary assembly and is highly heterozygous (1.69%; supplementary fig. S1, Supplementary Material online).

**Table 1:** Summary statistics for the gfSpoDele1 assembly and annotation

	Nuclear genome	Mitogenome
Number of contigs	11	1
Total size (bp)	30,870,404	112,912
N50 (Mbp)	3.05	…
Kmer QV	66.53	…
Number of protein-coding genes (high confidence/low confidence)	11,245 (9,409/1,836)	14 (35 ORFs, 19 intronic ORFs)
BUSCO completeness (assembly)	C: 94.4% [S: 93.8%, D: 0.6%], F: 0.8%, M: 4.8%, n: 4464	…
BUSCO completeness (annotation)	C: 98.6% [S: 97.7%, D: 0.9%], F: 0.1%, M: 1.3%, n: 4464	…

We predicted telomeres on both ends of 9 out of the total 11 pseudo-chromosomes, with the remaining 2 contigs (SUPER_9 and SUPER_11) having a telomere at one end (Fig. 1b). A total of 11 chromosomes was slightly lower than the number that has been previously reported for other *Polyporales* species assembled to chromosome level, which ranges from 12 to 14 (Chen et al. 2012, 2022; Wright et al. 2022; Ma et al. 2024), although this was explained by 3 chromosomal “fusions” that were flagged when we examined synteny relative to the *Laetiporus sulphureus* gfLaeSulp1 assembly (Fig. 1d). Each of these fusion events was supported following curation and manual checking of read alignments.

In addition to the nuclear genome assembly, we assembled and annotated the mitochondrial genome (Fig. 1c), which was a single circular contig 112.9 Kbp in length and included all 14 core protein-coding genes typically present in fungal mitogenomes (Sandor et al. 2018).

### Gene and CAZyme Content

Gene models were generated using REAT (Robust Eukaryotic Annotation Toolkit; https://github.com/EI-CoreBioinformatics/reat) and functionally annotated with AHRD (https://github.com/groupschoof/AHRD; supplementary file S1, Supplementary Material online). We compared gene content across species with available genome annotations within the *Polyporales* “residual” clade as defined by Justo et al. (2017), including *L. sulphureus* as an outgroup (supplementary table S1, Supplementary Material online). This first involved inference of a species tree from orthologous genes using OrthoFinder (Emms and Kelly 2019), where we found that the divergence of families in the phylogenomic tree corresponded with previous multilocus phylogenetic analysis within the order (Justo et al. 2017). We additionally predicted carbohydrate-active enzyme (CAZyme) genes using run_dbcan (Zheng et al. 2023; supplementary file S2, Supplementary Material online), as CAZymes are a major component of the gene repertoire necessary for white-rot (Hage et al. 2021). The total number of genes and CAZymes was slightly lower than either the closest available relative within *Cerrenaceae*, *Cerrena unicolor*, or *Panus rudis* in the sister family of *Panaceae* (Fig. 1e). The proportions of CAZymes known to act on various substrates were similar across all taxa, however.

## Conclusion

Here, we present the first near-complete, annotated genome assembly for the wood-decaying bracket fungus *S. occarium* (previously *Sp. delectans*). The first representative for Kingdom *Fungi* under the ERGA initiative, this new high-quality genome resource will enable further exploration of the genetic basis of saprotrophy in an ecologically and chemically important lineage of fungi, the *Polyporales*.

## Materials and Methods

### Sample Collection and Isolation

The *S. occarium* strain sequenced here was collected as a fresh basidiome growing on dead wood of *Quercus cerris*, on 2005 October 20 at the Rendez-vous nature monument in Czech Republic (Valtice, Břeclav, South Moravian region, 48.7499006N, 16.7939872E). This is the same locality from which the epitype of *S. occarium* was collected (Miettinen et al. 2023). The basidiome was placed on a petri dish of 2% malt extract agar, and the resulting culture was deposited in the culture collection of MENDELU (accession MUcc 838) and simultaneously kept in the Culture Collection of Basidiomycetes of the Institute of Microbiology, Prague (accession CCBAS136). Its identity was confirmed using phylogenetic analysis by Tomšovský (2012), under the previous name of *Sp. delectans*.

### Nucleic Acid Isolation and Sequencing

#### DNA and RNA Extractions

Fresh mycelia were obtained after 2 week of stationary cultivation in 2% malt extract liquid media (24 °C, dark), washed in deionized water, hand-squeezed, and stored at −80 °C prior to DNA or RNA extraction. High molecular weight (HMW) DNA was isolated in 8 aliquots, each consisting of up to 200 mg of frozen biomass, using a modified phenol–chloroform extraction (Sagova-Mareckova et al. 2008). Frozen biomass was homogenized in liquid nitrogen to a fine powder. All vortexing steps in the workflow were replaced by 50× repeated inversions of tubes to prevent DNA fragmentation. DNA was eluted in 10 mM Tris-Cl, pH 8.5, and stored at −80 °C. RNA was extracted in 6 aliquots of at least 50 mg of mycelia using the Quick RNA Fungal/Bacterial Miniprep kit (Zymo Research) according to the manufacturer’s protocol, excepting DNAse treatment, which was carried out separately using the Turbo DNA-free kit (Invitrogen) and stored at −80 °C. The quality of the isolated DNA/RNA was checked with gel electrophoresis, where samples with HMW DNA or RNA showing 3 separate bands for RNA subunits were considered acceptable.

#### PacBio HiFi Genome Sequencing

Four HWM DNA extractions were combined to construct a PacBio HiFi library at the Earlham Institute, Norwich, UK using the SMRTbell Express Template Prep Kit 2.0 (PacBio, P/N 100-983-900). In total, 18 µg of HWM DNA was manually sheared with the Megaruptor 3 instrument (Diagenode, P/N B06010003) according to the operations manual. After shearing, the sample underwent AMPure PB bead (PacBio, P/N 100-265-900) purification and concentration before undergoing library preparation using the SMRTbell Express Template Prep Kit 2.0 (PacBio, P/N 100-983-900). The library was prepared according to the HiFi protocol v03 (PacBio, P/N 101-853-100) and the final library was size fractionated using the SageELF system (Sage Science, P/N ELF0001) and a 0.75% cassette (Sage Science, P/N ELD7510). The library was quantified by fluorescence (Invitrogen Qubit 3.0, P/N Q33216) and the size of the library fractions was estimated from a smear analysis performed on the FEMTO Pulse System (Agilent, P/N M5330AA). The loading calculations for sequencing were completed using the PacBio SMRTLink Binding Calculator 10.1. Sequencing primer v2 was annealed to the adapter sequence of the HiFi library. The library was bound to the sequencing polymerase with the Sequel II Binding Kit v2.0 (PacBio, P/N 101-842-900). Calculations for primer and polymerase binding ratios were kept at default values for the library type. Sequel II DNA internal control 1.0 was spiked into the library at the standard concentration prior to sequencing. The sequencing chemistry used was Sequel II Sequencing Plate 2.0 (PacBio, P/N 101-820-200) and the Instrument Control Software v10.1.0.125432. The library was sequenced on the Sequel IIe on 1 Sequel II SMRTcell 8 M. The parameters for sequencing were diffusion loading, a 30-h movie, a 4-h pre-extension time, a 2-h immobilization, and 70 pM on plate loading concentration.

#### PacBio Iso-Seq

One PacBio Iso-Seq library was constructed starting from 315 ng of total RNA according to the guidelines laid out in the Iso-Seq protocol v02 (PacBio, 101-763-800), using SMRTbell express template prep kit 2.0 (PacBio, 102-088-900). Please see the Supplementary material for full details. The sequencing chemistry used was Sequel II Sequencing Plate 2.0 (PacBio, 101-820-200) and the Instrument Control Software v10.1.0.119549. The Iso-Seq library was sequenced on the Sequel IIe instrument with 1 Sequel II SMRTcell 8 M cell. The parameters for sequencing were diffusion loading, a 30-h movie, a 2-h immobilization time, a 2-h preextension time, and an 80 pM on plate loading concentration.

#### Illumina RNA-Seq

One RNA-Seq library was constructed using the NEBNext Ultra II RNA Library prep for Illumina kit (NEB#E7760L), NEBNext Poly(A) mRNA magnetic isolation module (NEB#7490), and NEBNext Multiplex Oligos for Illumina (E6440S) at a concentration of 10 µM. Please see the Supplementary material for full details. The library was loaded onto a v1.5 NovaSeq SP flow cell using the NovaSeq Xp flow cell dock. The flow cell was then loaded onto the NovaSeq 6000 along with a NovaSeq 6000 v1.5 SP cluster cartridge, a buffer cartridge, and a 300-cycle SBS cartridge (Illumina). The NovaSeq was run with NVCS v1.7.5 and RTA v3.4.4, and was set up to sequence 150 bp paired-end reads. The data were demultiplexed and converted to fastq using bcl2fastq2.

### Genome Assembly

PacBio HiFi reads were randomly subsampled to ∼100× coverage and assembled using hifiasm v0.16.1 (Cheng et al. 2021). Alternative haplotypes were removed using purge_dups v1.0.1 (Guan et al. 2020). The assembly was manually curated by loading it into the Gap5 sequence editor (Bonfield and Whitwham 2010), and each contig’s integrity was assessed. Four breaks were made due to erroneously assembled reads at contig ends, and 1 join was made between 2 contigs based on repeat analysis and genome synteny with the *Trametes hirsuta* reference genome (accession GCA_001302255.2).

Assembly completeness was estimated using BUSCO v5.4.7 with the Polyporales_odb10 dataset (Manni et al. 2021). The assembly consensus quality value (QV) was estimated using Merqury v1.3 (Rhie et al. 2020). Telomeric repeats (TTAGGG) at the ends of fragments were identified using tidk v0.2.31 (Brown 2023), with at least 5 repeats required for positive telomere prediction. The mitochondrial genome was assembled separately using IPA (https://github.com/PacificBiosciences/pbipa).

### Genome Annotation

Annotation of repetitive elements was performed using the EI-Repeat pipeline v1.1.0 (https://github.com/EI-CoreBioinformatics/eirepeat), which masked the genome assembly using RepeatMasker v4.0.7 (Smit et al. 2015) with a repeat library from RepBase and a *de novo* library of repeats constructed using RepeatModeler v1.0.11 (Smit and Hubley 2015).

Gene models were annotated using REAT (https://github.com/EI-CoreBioinformatics/reat) and Minos (https://github.com/EI-CoreBioinformatics/minos), incorporating transcript assemblies from Illumina RNA-Seq and PacBio Iso-Seq data, alignment of protein sequences from related species, and evidence-guided gene prediction with AUGUSTUS (Stanke and Morgenstern 2005). Please see the Supplementary material for full details. Gene models were functionally annotated using EI-FunAnnot pipeline v1.3 (https://github.com/EI-CoreBioinformatics/eifunannot) utilizing AHRD v3.3.3 (https://github.com/groupschoof/AHRD) with hits against fungal proteins from both Swiss-Prot and TrEMBL (downloaded 2022 June 15; The UniProt Consortium 2021) generated with BLAST v2.6.0 (Camacho et al. 2009) as well as results from InterProScan v5.22.61 (Jones et al. 2014).

The mitochondrial genome was annotated using MFannot v1.35 (Lang et al. 2023) and visualized using OGDRAW (Greiner et al. 2019).

Assembly and annotation data were submitted to the European Nucleotide Archive using COPO (Shaw et al. 2020).

### Phylogenomics and Comparative Genomics

Assessment of syntenic conservation and collinearity with the chromosome-scale genome assembly of *L. sulphureus* gfLaeSulp1 (Wright et al. 2022) was performed using MCScanX v1.1 (Wang et al. 2012) and visualized using SynVisio (Bandi and Gutwin 2020).

For phylogenomic reconstruction of the *Polyporales* “residual” clade *sensu* Justo et al. (2017), we inferred orthologous gene families by running OrthoFinder v2.5.4 (Emms and Kelly 2019) on the *S. occarium* proteome alongside proteomes from other residual *Polyporales* taxa with available data and *L. sulphureus* as an outgroup (supplementary table S1, Supplementary Material online). CAZyme genes for all taxa were predicted using run_dbcan v4 (Huang et al. 2018; Zhang et al. 2018; Zheng et al. 2023) and visualized alongside the STAG species tree produced by OrthoFinder.

## Supplementary Material

evaf003_Supplementary_Data

## Data Availability

Genome assembly and annotation data are available in the European Nucleotide Archive under BioProject PRJEB75241 and accession GCA_964035595.1.

## References

[evaf003-B1] Bandi V, Gutwin C. Interactive exploration of genomic conservation. In: Proceedings of the 46th graphics interface conference. Canadian Human-Computer Communications Society: University of Toronto; 2020.

[evaf003-B2] Bonfield JK, Whitwham A. Gap5—editing the billion fragment sequence assembly. Bioinformatics. 2010:26(14):1699–1703. 10.1093/bioinformatics/btq268. 20513662 PMC2894512

[evaf003-B3] Brown M . 2023. A Telomere Identification toolKit (tidk). https://github.com/tolkit/telomeric-identifier.

[evaf003-B4] Camacho C, Coulouris G, Avagyan V, Ma N, Papadopoulos J, Bealer K, Madden TL. BLAST+: architecture and applications. BMC Bioinformatics. 2009:10(1):421. 10.1186/1471-2105-10-421. 20003500 PMC2803857

[evaf003-B5] Chen C-L, Li W-C, Chuang Y-C, Liu H-C, Huang C-H, Lo K-Y, Chen C-Y, Chang F-M, Chang G-A, Lin Y-L, et al Sexual crossing, chromosome-level genome sequences, and comparative genomic analyses for the medicinal mushroom *Taiwanofungus Camphoratus* (syn. *Antrodia Cinnamomea*, *Antrodia Camphorata*). Microbiol Spectr. 2022:10(1):e02032-21. 10.1128/spectrum.02032-21. 35196809 PMC8865532

[evaf003-B6] Chen S, Xu J, Liu C, Zhu Y, Nelson DR, Zhou S, Li C, Wang L, Guo X, Sun Y, et al Genome sequence of the model medicinal mushroom *Ganoderma lucidum*. Nat Commun. 2012:3(1):913. 10.1038/ncomms1923. 22735441 PMC3621433

[evaf003-B7] Cheng H, Concepcion GT, Feng X, Zhang H, Li H. Haplotype-resolved de novo assembly using phased assembly graphs with hifiasm. Nat Methods. 2021:18(2):170–175. 10.1038/s41592-020-01056-5. 33526886 PMC7961889

[evaf003-B8] Dahlberg A, Mueller GM. Applying IUCN red-listing criteria for assessing and reporting on the conservation status of fungal species. Fungal Ecol. 2011:4(2):147–162. 10.1016/j.funeco.2010.11.001.

[evaf003-B9] EBP . 2023. *Report on Assembly Standards v5*. https://www.earthbiogenome.org/report-on-assembly-standards (Accessed April 9, 2024).

[evaf003-B10] Emms DM, Kelly S. OrthoFinder: phylogenetic orthology inference for comparative genomics. Genome Biol. 2019:20(1):238. 10.1101/466201. 31727128 PMC6857279

[evaf003-B11] GBIF . 2024. *Spongipellis delectans* (Peck) Murrill. https://www.gbif.org/species/2543646 (Accessed April 17, 2024).

[evaf003-B12] Greiner S, Lehwark P, Bock R. OrganellarGenomeDRAW (OGDRAW) version 1.3.1: expanded toolkit for the graphical visualization of organellar genomes. Nucleic Acids Res. 2019:47(W1):W59–W64. 10.1093/nar/gkz238. 30949694 PMC6602502

[evaf003-B13] Grienke U, Zöll M, Peintner U, Rollinger JM. European medicinal polypores—a modern view on traditional uses. J Ethnopharmacol. 2014:154(3):564–583. 10.1016/j.jep.2014.04.030. 24786572

[evaf003-B14] Guan D, McCarthy SA, Wood J, Howe K, Wang Y, Durbin R. Identifying and removing haplotypic duplication in primary genome assemblies. Bioinformatics. 2020:36(9):2896–2898. 10.1093/bioinformatics/btaa025. 31971576 PMC7203741

[evaf003-B15] Hage H, Miyauchi S, Virágh M, Drula E, Min B, Chaduli D, Navarro D, Favel A, Norest M, Lesage-Meessen L, et al Gene family expansions and transcriptome signatures uncover fungal adaptations to wood decay. Environ Microbiol. 2021:23(10):5716–5732. 10.1111/1462-2920.15423. 33538380 PMC8596683

[evaf003-B16] Hallenberg N, Ryberg M, Nilsson RH, Wood AR, Wu S-H. *Pseudolagarobasidium* (basidiomycota): on the reinstatement of a genus of parasitic, saprophytic, and endophytic resupinate fungi. Botany. 2008:86(11):1319–1325. 10.1139/B08-088.

[evaf003-B17] Halme P, Kotiaho JS, Ylisirniö A-L, Hottola J, Junninen K, Kouki J, Lindgren M, Mönkkönen M, Penttilä R, Renvall P, et al Perennial polypores as indicators of annual and red-listed polypores. Ecol Indic. 2009:9(2):256–266. 10.1016/j.ecolind.2008.04.005.

[evaf003-B18] Huang L, Zhang H, Wu P, Entwistle S, Li X, Yohe T, Yi H, Yang Z, Yin Y. dbCAN-seq: a database of carbohydrate-active enzyme (CAZyme) sequence and annotation. Nucleic Acids Res. 2018:46(D1):D516–D521. 10.1093/nar/gkx894. 30053267 PMC5753378

[evaf003-B19] IUCN . 2021. Re:wild and IUCN SSC become first global organizations to call for the recognition of fungi as one of three kingdoms of life critical to protecting and restoring Earth. https://www.iucn.org/news/species-survival-commission/202108/rewild-and-iucn-ssc-become-first-global-organizations-call-recognition-fungi-one-three-kingdoms-life-critical-protecting-and-restoring-earth (Accessed April 17, 2024).

[evaf003-B20] Jones P, Binns D, Chang H-Y, Fraser M, Li W, McAnulla C, McWilliam H, Maslen J, Mitchell A, Nuka G, et al InterProScan 5: genome-scale protein function classification. Bioinformatics. 2014:30(9):1236–1240. 10.1093/bioinformatics/btu031. 24451626 PMC3998142

[evaf003-B21] Jönsson MT, Edman M, Jonsson BG. Colonization and extinction patterns of wood-decaying fungi in a boreal old-growth *Picea abies* forest. J Ecol. 2008:96(5):1065–1075. 10.1111/j.1365-2745.2008.01411.x.

[evaf003-B22] Justo A, Miettinen O, Floudas D, Ortiz-Santana B, Sjökvist E, Lindner D, Nakasone K, Niemelä T, Larsson K-H, Ryvarden L, et al A revised family-level classification of the *Polyporales* (*Basidiomycota*). Fungal Biol. 2017:121(9):798–824. 10.1016/j.funbio.2017.05.010. 28800851

[evaf003-B23] Lang BF, Beck N, Prince S, Sarrasin M, Rioux P, Burger G. Mitochondrial genome annotation with MFannot: a critical analysis of gene identification and gene model prediction. Front Plant Sci. 2023:14:1222186. 10.3389/fpls.2023.1222186. 37469769 PMC10352661

[evaf003-B24] Lewin HA, Robinson GE, Kress WJ, Baker WJ, Coddington J, Crandall KA, Durbin R, Edwards SV, Forest F, Gilbert MTP, et al Earth BioGenome project: sequencing life for the future of life. Proc Natl Acad Sci U S A. 2018:115(17):4325–4333. 10.1073/pnas.1720115115. 29686065 PMC5924910

[evaf003-B25] Lonsdale D, Pautasso M, Holdenrieder O. Wood-decaying fungi in the forest: conservation needs and management options. Eur J Forest Res. 2008:127(1):1–22. 10.1007/s10342-007-0182-6.

[evaf003-B26] Ma J-X, Wang H, Jin C, Ye Y-F, Tang L-X, Si J, Song J. Whole genome sequencing and annotation of *Daedaleopsis sinensis*, a wood-decaying fungus significantly degrading lignocellulose. Front Bioeng Biotechnol. 2024:11:1325088. 10.3389/fbioe.2023.1325088. 38292304 PMC10826855

[evaf003-B27] Manni M, Berkeley MR, Seppey M, Simão FA, Zdobnov EM. BUSCO update: novel and streamlined workflows along with broader and deeper phylogenetic coverage for scoring of eukaryotic, prokaryotic, and viral genomes. Mol Biol Evol. 2021:38(10):4647–4654. 10.1093/molbev/msab199. 34320186 PMC8476166

[evaf003-B28] May TW, Cooper JA, Dahlberg A, Furci G, Minter DW, Mueller GM, Pouliot A, Yang Z. Recognition of the discipline of conservation mycology. Conserv Biol. 2018:33(3):733–736. 10.1111/cobi.13228. 30264893

[evaf003-B29] Mc Cartney AM, Formenti G, Mouton A, De Panis D, Marins LS, Leitão HG, Diedericks G, Kirangwa J, Morselli M, Salces-Ortiz J, et al The European reference genome atlas: piloting a decentralised approach to equitable biodiversity genomics. NPJ Biodivers. 2024:3:28. 10.1038/s44185-024-00054-6. 39289538 PMC11408602

[evaf003-B30] Miettinen O, Vlasák J, Larsson E, Vlasák J, Seelan JSS, Levicky Q, Larsson K-H, Spirin V. A revised genus-level classification for *Cerrenaceae* (*Polyporales, Agaricomycetes*). Fungal Syst Evol. 2023:12(1):271–322. 10.3114/fuse.2023.12.14. 38455955 PMC10918759

[evaf003-B31] Miller R, Lodge D. Fungal responses to disturbance: agriculture and forestry. In: Kubicek CP, Druzhinina IS, editors. Environmental and microbial relationships. The mycota, A comprehensive treatise on fungi as experimental systems for basic and applied research, Vol. IV. Berlin: Springer-Verlag; 2007. p. 50–52.

[evaf003-B32] Mueller GM, Dahlberg A, Krikorev M. Bringing fungi into the conservation conversation: the global fungal red list initiative. Fungal Conserv. 2014:4:12–16. 10.1017/s0953756202226659.

[evaf003-B33] Prescott TAK, Hill R, Mas-Claret E, Gaya E, Burns E. Fungal drug discovery for chronic disease: history, new discoveries and new approaches. Biomolecules. 2023:13(6):986. 10.3390/biom13060986. 37371566 PMC10296638

[evaf003-B34] Rhie A, Walenz BP, Koren S, Phillippy AM. Merqury: reference-free quality, completeness, and phasing assessment for genome assemblies. Genome Biol. 2020:21(1):245. 10.1186/s13059-020-02134-9. 32928274 PMC7488777

[evaf003-B35] Sagova-Mareckova M, Cermak L, Novotna J, Plhackova K, Forstova J, Kopecky J. Innovative methods for soil DNA purification tested in soils with widely differing characteristics. Appl Environ Microbiol. 2008:74(9):2902–2907. 10.1128/AEM.02161-07. 18344341 PMC2394906

[evaf003-B36] Sandor S, Zhang Y, Xu J. Fungal mitochondrial genomes and genetic polymorphisms. Appl Microbiol Biotechnol. 2018:102(22):9433–9448. 10.1007/s00253-018-9350-5. 30209549

[evaf003-B37] Shaw F, Etuk A, Minotto A, Gonzalez-Beltran A, Johnson D, Rocca-Serra P, Laporte M-A, Arnaud E, Devare M, Kersey P, et al COPO: a metadata platform for brokering FAIR data in the life sciences. F1000Res. 2020:9:495. 10.12688/f1000research.23889.1.

[evaf003-B38] Smit A, Hubley R. 2015. RepeatModeler Open-1.0. http://www.repeatmasker.org.

[evaf003-B39] Smit A, Hubley R, Green P. 2015. RepeatMasker Open-4.0. http://www.repeatmasker.org.

[evaf003-B40] Stanke M, Morgenstern B. AUGUSTUS: a web server for gene prediction in eukaryotes that allows user-defined constraints. Nucleic Acids Res. 2005:33(Web Server):W465–W467. 10.1093/nar/gki458. 15980513 PMC1160219

[evaf003-B41] The UniProt Consortium . UniProt: the universal protein knowledgebase in 2021. Nucleic Acids Res. 2021:49(D1):D480–D489. 10.1093/nar/gkaa1100. 33237286 PMC7778908

[evaf003-B42] Tomšovský M . Delimitation of an almost forgotten species *Spongipellis litschaueri* (polyporales, basidiomycota) and its taxonomic position within the genus. Mycol Progress. 2012:11(2):415–424. 10.1007/s11557-011-0756-z.

[evaf003-B43] Větrovský T, Morais D, Kohout P, Lepinay C, Algora C, Awokunle Hollá S, Bahnmann BD, Bílohnědá K, Brabcová V, D'Alò F, et al GlobalFungi, a global database of fungal occurrences from high-throughput-sequencing metabarcoding studies. Sci Data. 2020:7(1):228. 10.1038/s41597-020-0567-7. 32661237 PMC7359306

[evaf003-B44] Wang Y, Tang H, Debarry JD, Tan X, Li J, Wang X, Lee T-h, Jin H, Marler B, Guo H, et al MCScanX: a toolkit for detection and evolutionary analysis of gene synteny and collinearity. Nucleic Acids Res. 2012:40(7):e49. 10.1093/nar/gkr1293. 22217600 PMC3326336

[evaf003-B45] Wright R, Woof K, Douglas B, Gaya E. The genome sequence of the chicken of the woods fungus, *Laetiporus sulphureus* (bull.) murrill, 1920. Wellcome Open Res. 2022:7:83. 10.12688/wellcomeopenres.17750.1. 37975019 PMC10652036

[evaf003-B46] Zhang H, Yohe T, Huang L, Entwistle S, Wu P, Yang Z, Busk PK, Xu Y, Yin Y. dbCAN2: a meta server for automated carbohydrate-active enzyme annotation. Nucleic Acids Res. 2018:46(W1):W95–W101. 10.1093/nar/gky418. 29771380 PMC6031026

[evaf003-B47] Zheng J, Ge Q, Yan Y, Zhang X, Huang L, Yin Y. dbCAN3: automated carbohydrate-active enzyme and substrate annotation. Nucleic Acids Res. 2023:51(W1):W115–W121. 10.1093/nar/gkad328. 37125649 PMC10320055

[evaf003-B48] Zíbarová L, Kolényová M, Tejklová T, Zehnálek P. Red list of fungi (macromycetes) of the Czech republic. Příroda. 2024:46:1–192. https://www.priroda.nature.cz/index.php/priroda/issue/view/17.

