# Supplementary material for: Nuclear and Mitochondrial Genome Assemblies for the Endangered Wood-Decaying Fungus Somion occarium
Source: Genome Biol Evol. 2025 Jan 10;17(1):evaf003. doi: 10.1093/gbe/evaf003 (PMC11783313; doi:10.1093/gbe/evaf003)
Supplement: evaf003_Supplementary_Data [file evaf003_supplementary_data.zip › Supplementary Material.pdf]

SUPPLEMENTARY MATERIAL - Nuclear and mitochondrial genome assemblies for the endangered wood-decaying fungus *Somion occarium*

PacBio Iso-Seq

One PacBio Iso-Seq library was constructed starting from 315ng of total RNA. Reverse transcription and cDNA synthesis was performed using NEBNext Single Cell/Low Input cDNA Synthesis & Amplification Module (NEB, E6421). The cDNA sample was amplified with a barcoded primer for a total of 12 cycles. The library was prepared according to the guidelines laid out in the Iso-Seq protocol v02 (PacBio, 101-763-800), using SMRTbell express template prep kit 2.0 (PacBio, 102-088-900). The final library was quantified using a Qubit Fluorometer 3.0 (Invitrogen) and sized using the Bioanalyzer HS DNA chip (Agilent). The loading calculations for the Iso-Seq library were completed using the PacBio SMRTlink Binding Calculator v10.1. Sequencing primer v4 was annealed to the Iso-Seq library and complexed to the sequencing polymerase with the Sequel II binding kit v2.1 (PacBio, 101-843-000). Calculations for primer to template were 20X and polymerase to template binding ratios were 15X. Sequencing internal control complex 1.0 (PacBio, 101-717-600) was spiked into the final complex preparation at a standard concentration before sequencing. The sequencing chemistry used was Sequel II Sequencing Plate 2.0 (PacBio, 101-820-200) and the Instrument Control Software v10.1.0.119549. The Iso-Seq library was sequenced on the Sequel IIe instrument with one Sequel II SMRTcell 8M cell. The parameters for sequencing were diffusion loading, 30-hour movie, 2-hour immobilisation time, 2-hour pre-extension time, and 80pM on plate loading concentration.

Illumina RNA-Seq

One RNA-Seq library was constructed using the NEBNext Ultra II RNA Library prep for Illumina kit (NEB#E7760L), NEBNext Poly(A) mRNA Magnetic Isolation Module (NEB#7490) and NEBNext Multiplex Oligos for Illumina (E6440S) at a concentration of 10µM. Total RNA (1µg) was purified to extract mRNA with a Poly(A) mRNA Magnetic Isolation Module. Isolated mRNA was then fragmented for 12 minutes at 94°C and converted to cDNA. NEBNext adaptors were ligated to end-repaired, dA-tailed DNA. The ligated products were purified using Beckman Coulter AMPure XP beads (A63882) and enriched with 10 cycles of PCR (30 secs at 98°C, 10 cycles of: 10 secs at 98°C, 75 secs at 65°C, 5 mins at 65°C, final hold at 4°C). The size of the resulting library was determined using Agilent High Sensitivity DNA Kit (Agilent, 5067-4626) and the concentration quantified by qPCR using Kapa Library Quantification Kit (Roche Diagnostics, 7960204001). The final library was diluted down to 0.5 nM using EB (10mM Tris pH8.0) in a volume of 18µl before spiking in 1% Illumina phiX Control v3. This was denatured by adding 4µl 0.2N NaOH and incubating at room temperature for 8 mins, after which it was neutralised by adding 5µl 400mM tris pH 8.0. A master mix of EPX1, EPX2, and EPX3 from Illumina's Xp 2-lane kit v1.5 (Illumina, 20043130) was made and 63µl added to the denatured pool leaving 90µl at a concentration of 100pM. This was loaded onto a v1.5 NovaSeq SP flow cell using the NovaSeq Xp Flow Cell Dock. The flow cell was then loaded onto the NovaSeq 6000 along with an NovaSeq 6000 v1.5 SP cluster cartridge, buffer cartridge, and 300 cycle SBS cartridge (Illumina). The NovaSeq was run with NVCS v1.7.5 and RTA v3.4.4, and was set up to sequence 150bp paired-end reads. The data was demultiplexed and converted to fastq using bcl2fastq2.

REAT structural annotation workflow

The REAT Transcriptome workflow performed alignment of short-read RNA-Seq data using HISAT2 v2.2.1 (Kim et al. 2019) and PacBio Iso-Seq data with minimap2 v2.18-r1015 (Li 2021).

SUPPLEMENTARY MATERIAL - Nuclear and mitochondrial genome assemblies for the endangered wood-decaying fungus *Somion occarium*

High-confidence splice junctions were identified using Portcullis v.1.2.3 (Mapleson et al. 2018). Alignments from short-reads were assembled using StringTie v2.1.5 (Kovaka et al. 2019) and Scallop v0.10.5 (Shao and Kingsford 2017), while alignments from Iso-Seq data were assembled using StringTie. A consolidated set of transcriptome derived gene models was generated using Mikado v2.3.3 (https://github.com/EI-CoreBioinformatics/mikado; Venturini et al. 2018). The REAT Homology workflow was used to align protein sequences from 23 Polyporales species (supplementary table S2) against the *S. occarium* genome assembly. Proteins were aligned using Spaln v2.4.7 (Iwata and Gotoh 2012) and filtered to remove misaligned proteins. The aligned proteins were clustered into loci and a consolidated set of gene models was derived via Mikado. The REAT Prediction workflow was used to generate a set of evidence guided gene predictions by training AUGUSTUS (Stanke and Morgenstern 2005) with high confidence gene models from the Transcriptome and Homology workflows. Four alternative AUGUSTUS runs were performed with varying weightings of evidence, which were provided to EVidenceModeler (Haas et al. 2008) along with the transcriptome and protein evidence to generate consensus gene structures. The final set of gene models was selected using Minos from the output of the REAT Homology and Transcriptome runs, EVidenceModeler, and the AUGUSTUS runs based on metrics derived from the protein, transcript, and expression data.

Haas BJ et al. 2008. Automated eukaryotic gene structure annotation using EVidenceModeler and the Program to Assemble Spliced Alignments. Genome Biology. 9:R7. doi: 10.1186/gb-2008-9-1-r7.

Iwata H, Gotoh O. 2012. Benchmarking spliced alignment programs including Spaln2, an extended version of Spaln that incorporates additional species-specific features. Nucleic Acids Research. 40:e161. doi: 10.1093/nar/gks708.

Kim D, Paggi JM, Park C, Bennett C, Salzberg SL. 2019. Graph-based genome alignment and genotyping with HISAT2 and HISAT-genotype. Nat Biotechnol. 37:907–915. doi: 10.1038/s41587-019-0201-4.

Kovaka S et al. 2019. Transcriptome assembly from long-read RNA-seq alignments with StringTie2. Genome Biology. 20:278. doi: 10.1186/s13059-019-1910-1.

Li H. 2021. New strategies to improve minimap2 alignment accuracy. Bioinformatics. 37:4572–4574. doi: 10.1093/bioinformatics/btab705.

Mapleson D, Venturini L, Kaithakottil G, Swarbreck D. 2018. Efficient and accurate detection of splice junctions from RNA-seq with Portcullis. GigaScience. 7:giy131. doi: 10.1093/gigascience/giy131.

Shao M, Kingsford C. 2017. Accurate assembly of transcripts through phase-preserving graph decomposition. Nat Biotechnol. 35:1167–1169. doi: 10.1038/nbt.4020.

Stanke M, Morgenstern B. 2005. AUGUSTUS: a web server for gene prediction in eukaryotes that allows user-defined constraints. Nucleic Acids Research. 33:W465–W467. doi: 10.1093/nar/gki458.

Venturini L, Caim S, Kaithakottil GG, Mapleson DL, Swarbreck D. 2018. Leveraging multiple transcriptome assembly methods for improved gene structure annotation. GigaScience. 7:giy093. doi: 10.1093/gigascience/giy093.

Supplementary Table S1 Details of strains used in comparative analyses.

Species	Strain	Accession	Reference
Steccherinum ochraceum	LE-BIN 3174	GCA_004332605.1	(Moiseenko et al. 2020)
Antrodiella citronella	DSM 108506	GCA_004802725.1	
Panus rudis	PR-1116	GCA_022160315.1	(Hage et al. 2021)
Abortiporus biennis	CIRM-BRFM 1778	GCA_022606235.1	(Hage et al. 2021)
Physisporinus lineatus	VT162	GCA_027627245.1	
Phlebia brevispora	MPL23	GCA_027627325.1	
Laetiporus sulphureus	93-53	GCF_001632365.1	(Nagy et al. 2016)
Cerrena unicolor	Cerun2	https://mycocosm.jgi.doe.gov/Cerun2/Cerun2.home.html	

Hage H et al. 2021. Gene family expansions and transcriptome signatures uncover fungal adaptations to wood decay. Environmental Microbiology. 23:5716–5732. doi: 10.1111/1462-2920.15423.

Moiseenko K et al. 2020. Data on the genome analysis of the wood-rotting fungus *Steccherinum ochraceum* LE-BIN 3174. Data in Brief. 29:105169. doi: 10.1016/j.dib.2020.105169.

Nagy LG et al. 2016. Comparative Genomics of Early-Diverging Mushroom-Forming Fungi Provides Insights into the Origins of Lignocellulose Decay Capabilities. Mol Biol Evol. 33:959–970. doi: 10.1093/molbev/msv337.

Supplementary Table S2 List of species used for cross-species protein alignment for genome annotation.

Species	NCBI accession
Antrodiella citrinella	GCA_004802725.1
Cubamyces sp.	GCA_022385745.1
Daedalea quercina	GCA_001632345.1
Fibroporia radiculosa	GCF_000313525.1
Fomitopsis schrenkii	GCA_000344655.2
Ganoderma sinense	GCA_002760635.1
Gelatoporia subvermispora	GCA_000320605.2
Irpex rosettiformis	GCA_022160335.1
Laetiporus sulphureus	GCF_001632365.1
Lentinus tigrinus	GCA_003813185.1
Obba rivulosa	GCA_001687445.1
Panus rudis	GCA_022160315.1
Phanerochaete carnosa	GCF_000300595.1
Phlebiopsis gigantea	GCA_000832265.1
Pilatotrama ljubarskyi	GCA_022385805.1
Polyporus arcularius	GCA_004369055.1
Steccherinum ochraceum	GCA_004332605.1
Trametes cingulata	GCA_022385765.1
Trametes cinnabarina	GCA_000765035.1
Trametes coccinea	GCA_002092935.1
Trametes pubescens	GCA_001895945.1
Trametopsis cervina	GCA_022385755.1
Wolfiporia cocos	GCA_000344635.1

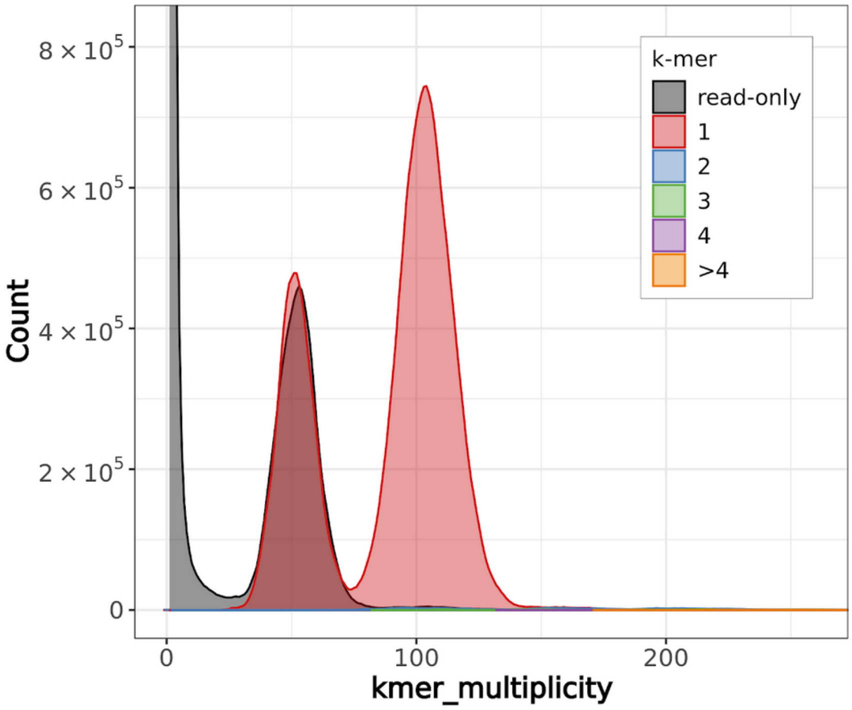

Supplementary Figure S1 Kmer multiplicity plot of sequencing reads indicating high levels of heterozygosity.
